# Novel inhibitors targeting Venezuelan equine encephalitis virus capsid protein identified using *In Silico* Structure-Based-Drug-Design

**DOI:** 10.1038/s41598-017-17672-9

**Published:** 2017-12-18

**Authors:** Sharon Shechter, David R. Thomas, Lindsay Lundberg, Chelsea Pinkham, Shih-Chao Lin, Kylie M. Wagstaff, Aaron Debono, Kylene Kehn-Hall, David A. Jans

**Affiliations:** 1 Shechter Computational Solutions, Andover, MA USA; 2 Nuclear Signaling Laboratory, Department of Biochemistry and Molecular Biology School of Biomedical Sciences, Monash University, Melbourne, Australia; 3 National Center for Biodefense and Infectious Diseases, School of Systems Biology, George Mason University, Manassas, VA USA; 4 Monash Institute of Pharmaceutical Sciences, Parkville, Victoria, Australia

## Abstract

Therapeutics are currently unavailable for Venezuelan equine encephalitis virus (VEEV), which elicits flu-like symptoms and encephalitis in humans, with an estimated 14% of cases resulting in neurological disease. Here we identify anti-VEEV agents using *in silico* structure-based-drug-design (SBDD) for the first time, characterising inhibitors that block recognition of VEEV capsid protein (C) by the host importin (IMP) α/β1 nuclear transport proteins. From an initial screen of 1.5 million compounds, followed by *in silico* refinement and screening for biological activity *in vitro*, we identified 21 hit compounds which inhibited IMPα/β1:C binding with IC_50_s as low as 5 µM. Four compounds were found to inhibit nuclear import of C in transfected cells, with one able to reduce VEEV replication at µM concentration, concomitant with reduced C nuclear accumulation in infected cells. Further, this compound was inactive against a mutant VEEV that lacks high affinity IMPα/β1:C interaction, supporting the mode of its antiviral action to be through inhibiting C nuclear localization. This successful application of SBDD paves the way for lead optimization for VEEV antivirals, and is an exciting prospect to identify inhibitors for the many other viral pathogens of significance that require IMPα/β1 in their infectious cycle.

## Introduction

Venezuelan equine encephalitis virus (VEEV) is an emerging pathogenic New World member of the genus *Alphavirus*, family *Togaviradae*. Along with viruses from the family *Flaviviridae*, togaviruses are extremely important human pathogens, being the most common cause of encephalitis^1^. Alphaviruses are enveloped positive-strand RNA viruses, which are arthropod borne, with mosquitoes being the usual vector^2^. Typical of most alphaviruses, the VEEV genome encodes 4 non-structural proteins (nsP1–4), which are critical for viral replication^3,4^, in addition to 5 structural proteins (capsid, E1-3 and 6 K)^5–7^, which are considered the primary targets for adaptive, antigen-specific immunity, and are also capable of interfering with host defense mechanisms^7–9^.

Capsid protein (C), found in the cytoplasm, nucleus and nuclear rim of infected host cells, is believed to have important roles in infection beyond encasing viral RNA^10,11^, including inhibiting the host antiviral response through a mechanism of blocking the host cell nucleocytoplasmic transport system^11,12^. Various RNA virus proteins are known to localize in the nucleus of infected host cells to antagonize the host antiviral response^13,14^, making viral protein nuclear import through the host cell nuclear transport machinery an attractive target for the development of antivirals^14–18^.

Entry into the nucleus through the nuclear envelope embedded nuclear pore complex (NPC) conventionally requires the recognition of a nuclear localization signal (NLS) within the cargo protein by members of the Importin (IMP) superfamily of nuclear transporters, of which there are multiple α and β types. “Classical” NLSs are recognized by the IMPα/β1 heterodimer, which mediates docking to and translocation through the NPC, a process that is essential to the host cell antiviral response (eg. through type I interferon - IFNα/β). VEEV C is believed to bind to IMPα, as well as to the host nuclear export protein exportin 1, leading to inhibition of host cell nuclear trafficking through either IMP/exportin 1 sequestration and/or blocking of the NPC itself^19^, thereby impacting host cell stress/innate immune responses^10^. This function of C has been mapped to amino acids (aa) 33-68, which contain an IMPα/β1-recognised NLS.

We previously used high throughput screening to identify Ivermectin and Mifepristone as inhibitors of IMPα/β1 binding to HIV-1 integrase^20^, subsequently showing that both had anti-HIV-1 activity;^18^ these compounds have since been found to be active against VEEV, through their ability to alter subcellular location of VEEV C^16^. The present study extends this work, applying *in silico* structure-based-drug-design (SBDD) for the first time to identify inhibitors of the IMPα/β1:C interaction. A number of these were validated in *in vitro* and cell-based assays, with one shown to reduce VEEV replication concomitant with reduced C nuclear accumulation in infected cells. The lead compound 1111684 was inactive against a VEEV C mutant lacking a functional NLS, consistent with the 1111684’s mechanism of action being to target IMPα/β1:C interaction. Our novel SBDD screening approach provides an exciting approach to identify inhibitors of viral pathogens that require IMPα/β1 for their infectious cycle, simultaneously detailing significant features of the IMPα/β1:NLS interaction that are critical for rational drug design to target it.

## Results

### *In silico* structure-based-drug-design for inhibitors of the IMPα/β1:C interaction

We set out to apply *in silico* SBDD screening to identify inhibitors of the IMPα/β1:VEEV C interaction using a peptidomimetics method^21–23^ to identify compounds that mimic protein-protein interactions, but have the advantage of being more drug-like. We based our work on PDB 3VE6, the only available crystal structure of the 12 amino acid C VEEV-NLS peptide bound to IMPα2 (Karyopherin subunit α2/KPNA2/Rch1), which has previously been shown to bind C^19^. Initial analysis involved identifying the minimum-NLS (Min-NLS) by reducing the length of the C NLS and assessing the impact thereof in docking experiments on the Glide score as well as the peptide’s conformation within the binding pocket of IMPα. In parallel, we identified key NLS-interacting residues in the IMPα binding pocket by running *in silico* alanine-mutagenesis scan docking experiments. We used the conformation of the Min-NLS within the full NLS peptide in the crystal structure, to define the minimal binding pocket (“Min-BP”) able to bind smaller NLS peptides as well as to build the docking grid, as described below.

### Identification of the Min-NLS for VEEV C

**Figure 1:**
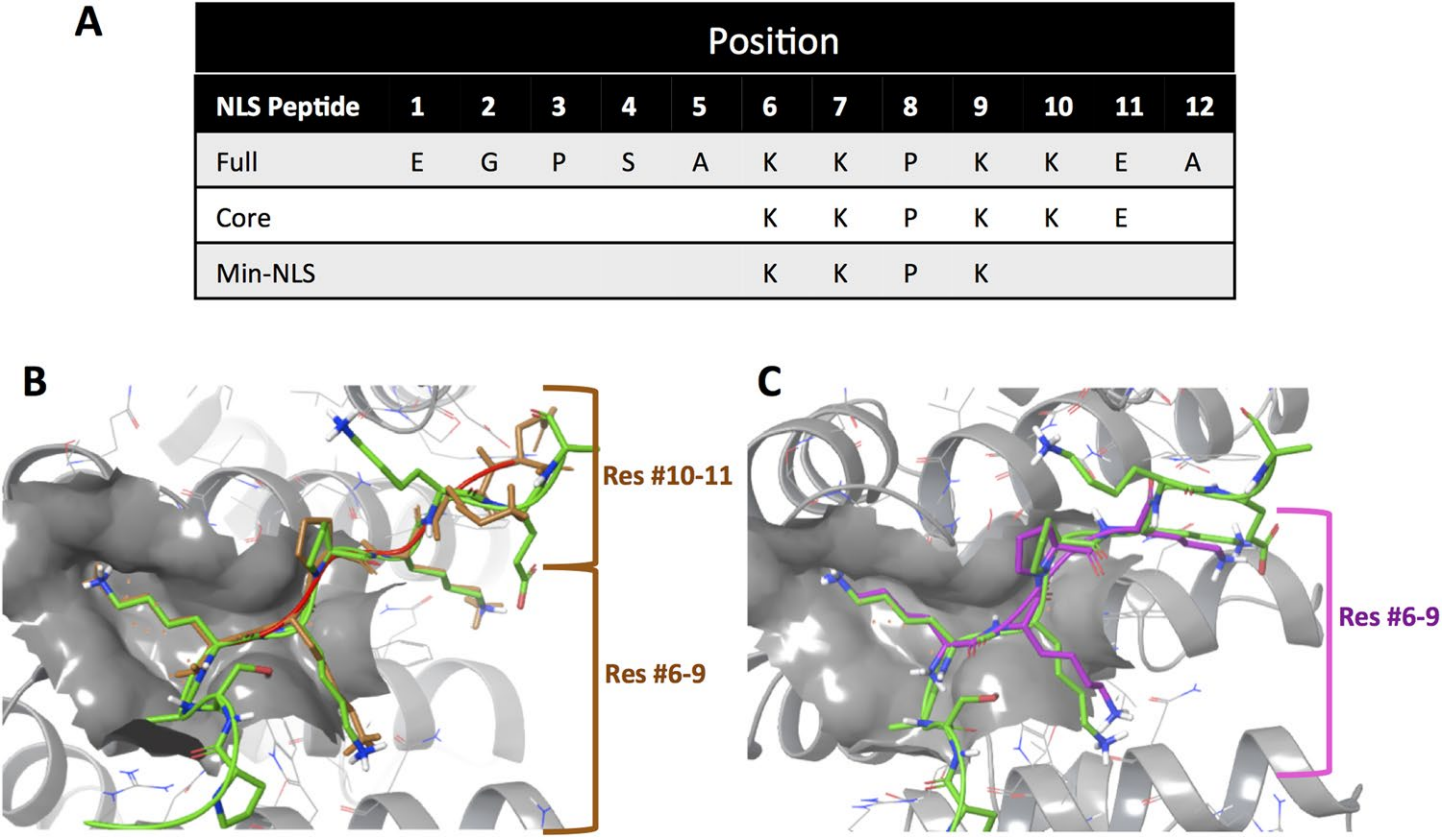
Definition of the Capsid NLS:IMPα interface. (**A**) Full, Core and Min-NLS sequences (single letter amino acid code). (**B**) Docked Core-NLS (brown) aligned on the full NLS (green) from the crystal structure of the VEEV NLS bound to the binding site of IMPα (grey) using Maestro (Schrodinger) and the PDB 3VE6 coordinates. Red and blue represent oxygen and nitrogen atoms respectively. (**C**) Docked Min-NLS (purple) aligned on the full NLS within the crystal structure of the VEEV NLS bound to the binding site of IMPα as per **B**.

K-K/R-X-K/R (single letter amino acid code), the proposed consensus core sequence of IMPα/β1 recognised NLSs^24–26^ served as the basis for selecting the ‘core’ of the C NLS (amino acids #6–11) (Table 1, Fig. 1A); conformers were generated using ConfGen (Schrodinger, Portland, OR, USA) and then docked using Glide (Schrodinger, Portland, OR, USA) to estimate the free energy of binding in the IMPα binding pocket. Notably, the best Glide scoring conformer for the docked Core-NLS structurally aligned well to residues #6-9 but not #10–11 of the crystal structure of the VEEV C-NLS bound to IMPα (Fig. 1B). Docking experiments were also performed for various smaller derivative peptides of the Core-NLS together with free energy calculations, to arrive ultimately at the Min-NLS (Table 1; Fig. 1C). The first K (position #6), which is known to be highly conserved in other NLS-bearing proteins that bind IMPα^27^ was critical, as its absence resulted in a significant increase in free binding energy as indicated by the Glide score, likely due to the loss of the salt bridge between the side-chains of the K, and D122 within the NLS-binding pocket of IMPα. Similarly, the second K (position #7) in the Min-NLS is important for binding as it utilises interactions with W114, W161, N118 and D200. Based on Tay *et al* ^27^., the P at position #8 appeared to be a key determinant in presenting the NLS in the correct conformation to bind IMPα. The third K (position #9) binds using hydrogen (H-) bonding with W72, N76, Q111 and W114, in addition to cation-π interactions with W72 and W114. We selected peptide #6-KKPK-#9 as the Min-NLS, since it agreed with the NLS core consensus, showed good superimposition with the full NLS, and most importantly, unlike the full NLS, was short enough to allow mimicry by a small molecule. Important in our considerations overall was that database analyses indicated that the KKPK sequence has been identified in only 13 human proteins (Supp. Table S1), implying that any compound targeting these residues should have only limited off-target effects, especially with only short term therapeutic use.

**Table 1:** Summary of free energy of NLS peptide-IMPα binding.

Peptide Sequences*	Glide score (kcal/mol)
EGPSAKKPKKEA (full C NLS^x^)	NA^#^
KKPKKE (residues 6–11; Core-NLS)	−11.836
KPKKE (residues 7–11)	−7.871
KKPK (residues 6–9; Min-NLS)	−9.642
KKP (residues 6–8)	−8.677

C NLS peptide and derivatives thereof were subjected to docking experiments with IMPα and the estimated free energy of binding calculated using the GLIDE software.

*The single letter amino acid code is used.

^X^From PDB 3VE6.

^#^Not able to be estimated due to the length of the full NLS.

### Identification of the key residues within the NLS-binding pocket of IMPα

**Figure 2:**
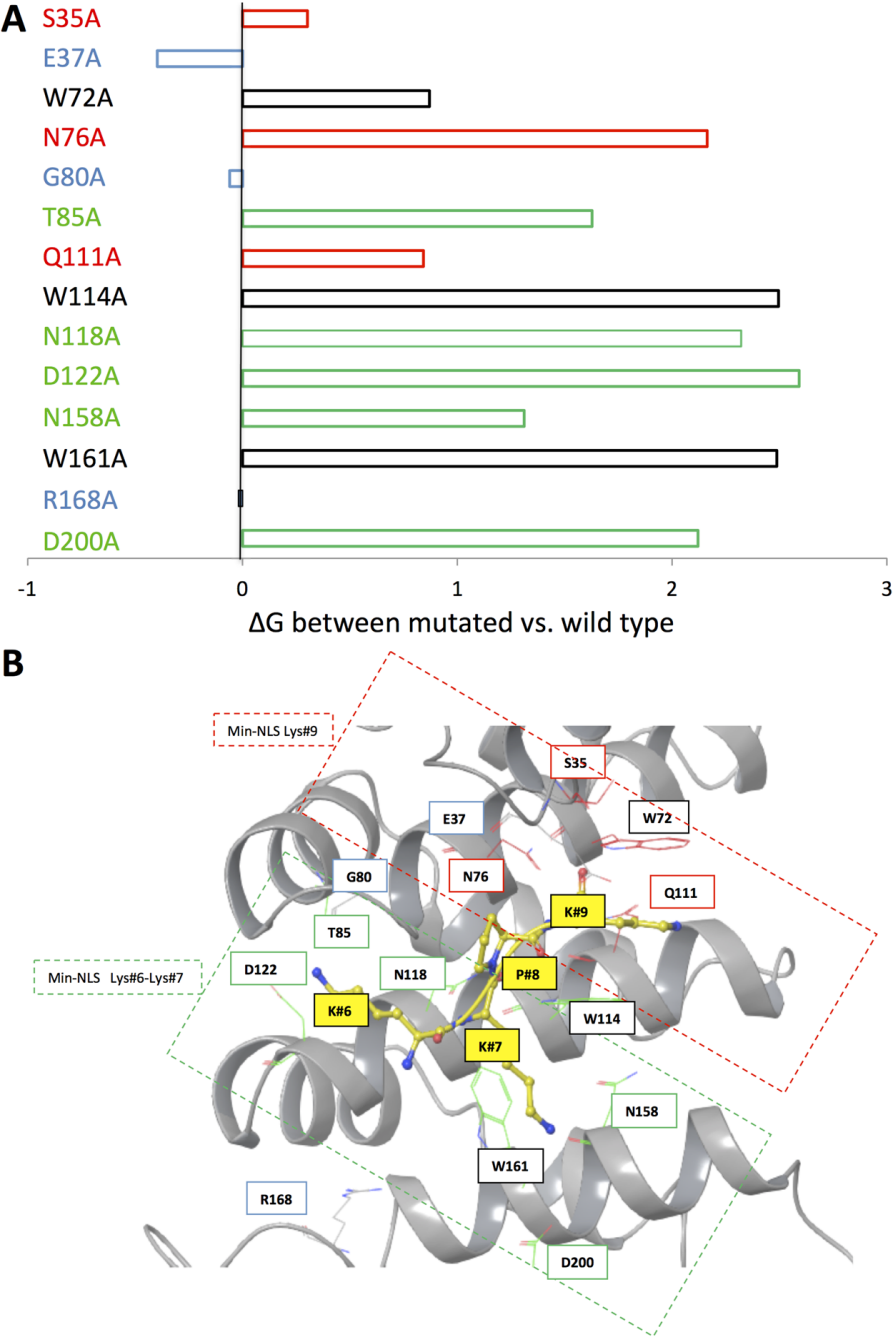
Delineation of the Min-NLS binding pocket of IMPα. (**A**) Difference in free energy estimations (ΔG) based on docking experiments for the indicated alanine substitutions of residues within the IMPα binding pocket less than 4 Å from the Min-NLS. For wild type IMPα as well as each alanine substitution, a new docking grid was created and a library of 62 Min-NLS conformations (ConfGen) was used for the docking experiment (Glide, Schrödinger). Based on the docking results, the best-aligned pose for docked Min-NLS consistent with its conformation in the PDB 3VE6 structure was selected to determine the ΔG. ΔG was calculated by using the docking score found with the mutated residue minus the calculated docking score of the wild type. Colour coding relates to “subcompartments” of the NLS binding pocket highlighted in B, where red denotes residues located in the “top” of the pocket, green denotes residues located in the “bottom” of the pocket, W residues appear in black, while blue denotes residues which may hinder compound binding. (**B**) Representation (Maestro, Schrödinger) of the IMPα binding pocket (grey) with bound Min-NLS residues (yellow), with their respective energy contribution color as seen in A; nitrogen and oxygen atoms are highlighted in blue and red respectively. The red dashed box highlights the “top” of the pocket (residues involved in interaction with the K at position #9 of the Min-NLS), whereas the green dashed box highlights the “bottom” of the pocket (residues involved in interacting with the K residues at positions #6 and 7).

**Figure 3:**
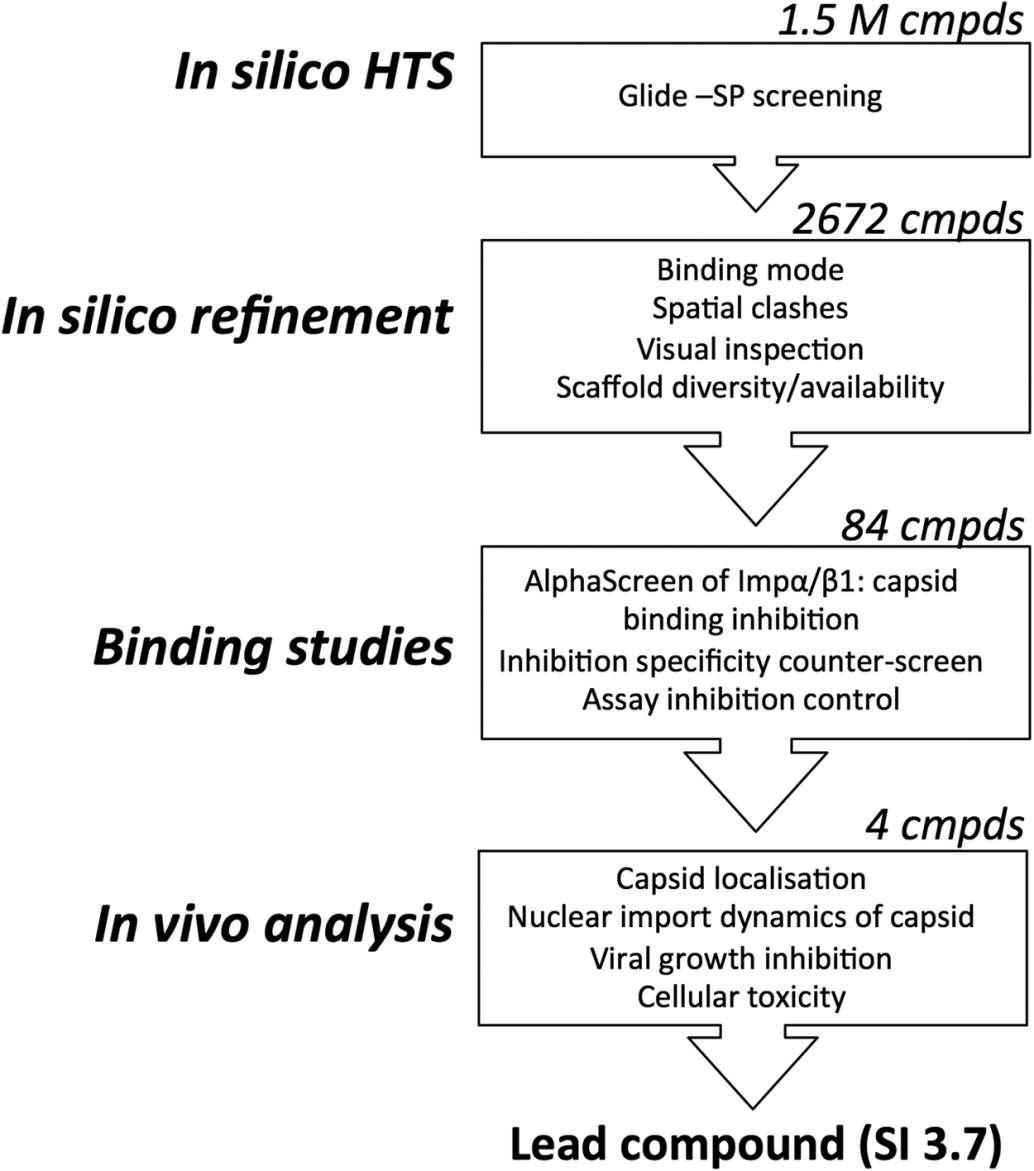
HTS Pipeline to identify inhibitors of C:IMPα binding. Glide (Schrodinger) was used to run a screen for inhibitors of C:IMPα binding from a curated library of 1.5 M compounds that was prepared using Ligprep (Schrodinger); Ligprep produces the low-energy 3D structures with correct chiralities. Consideration of the energy threshold (represented by the docking score), and binding mode and spatial clashes was used to prioirize the hit compounds (to 2672), followed by a scaffold diversity analysis (Schrodinger, Canvas) to enrich the final set. The final set of 84 commercially available compounds was subjected to binding studies examining their ability to inhibit the C:IMPα interaction. Four compounds ultimately progressed to *in vivo* analysis of their ability to alter C localization/dynamics and inhibition of VEEV replication.

We used free energy calculations to identify the key residues in the IMPα binding site that contribute most to NLS binding by performing *in silico* alanine-scan mutagenesis experiments. Specifically, each of the residues located in close proximity to the Min-NLS [<4 Å] in the binding site were computationally substituted with an A^28^, after which the free energy of binding was calculated in docking experiments. The free energy for each substitution was calculated and its value substracted from the free energy of the Min-NLS in the binding pocket of wild type IMPα to yield the ΔG value, an estimation of the contribution of each individual residue to Min-NLS interaction with IMPα (Fig. 2A). The D122 A substitution resulted in the biggest increase in binding energy, most likely due to the elimination of salt bridge interactions with K at position #6 in the Min-NLS, consistent with the *in silico* analysis above showing that the free energy binding goes up in the absence of this residue. Substantial changes in ΔG were also observed for alanine substitution of W161 and W114 (Fig. 2A), presumably due to effects on the hydrophobic interactions. Alanine substitution of N118, N158 and N76, all of which have been shown to interact with NLSs from viral proteins^27^, also increased the ΔG. In contrast, the E37A substitution decreased the free energy and thus potentially enhanced interaction, presumably due to the elimination of electrostatic repulsion between the negatively charged side chain of E37 and carbonyl group of the K at position #9. This information was used to help build the docking grid of the Min-NLS binding pocket on IMPα (Fig. 2B), which was used in the *in silico* screen for inhibitors (Fig. 3), as well as for subsequent stages of compound prioritisation for experimental validation (see below).

### *In silico* screen for inhibitors of IMPα/β1:C binding as anti-VEEV agents targeting C nuclear import

A set of 1.5 million compounds consisting of publicly available libraries and curated using Ligprep (Schrodinger, Portland, OR, USA) was subjected to a virtual screen meant to identify inhibitors of IMPα:C interaction as potential inhibitors of VEEV infection as outlined in Fig. 3. Briefly, the SBDD process involved large-scale docking using the crystal structure for mouse IMPα (3VE6) in association with *in silico* prediction and analysis as well as peptidomimetic principles for hit discovery. Each step was iterative, with each sequential iteration resulting in filtration of the library compounds used for docking; to assess the method’s performance at each stage of the screening process, random compounds from the surviving library were juxtaposed to the structure of the crystallized full-NLS peptide in 3VE6 to make sure that compounds proceeding to the next stage were well aligned and maintained the key binding interactions. Since we were looking for compounds smaller than the full-NLS peptide, we considered not only known interactions seen with the peptide (e.g. salt bridge interactions with D122) from the crystal structure, but also novel interactions (eg. hydrophobic interaction-like π-π interactions with W161, W114 and W72 within the binding pocket – Fig. 2).

Initial docking was performed using Glide-SP (standard precision) software (Schrodinger, Portland, OR, USA). Glide searches for favorable interactions between one or more ligand molecules (library of inhibitor compounds) and a receptor molecule (IMPα binding pocket). The shape and properties of IMPα in this case are represented on a grid by several different sets of fields that provide progressively more accurate scoring of the ligand poses. The highest 1% scoring compounds according to free energy minimization considerations were selected as the “Ranked set”, which was then analysed for the following properties: (a) good alignment with the crystallized Full-NLS peptide, (b) good occupancy of the binding pocket, and (c) lack of spatial clashes (overlap volume <4.5 Å) between the atoms of the compound and the protein target^29^.

All 2,672 compounds within the ranked set were considered candidates potentially worthy of testing for biological activity. We reduced this number by applying 2D similarity based clustering (Canvas, Schrodinger), which ensured a chemically diverse selected set of compounds. Clustering of compounds was based on structural similarity assessed using the multiple level atom neighborhoods method^30^. Compounds with Tanimoto cutoff values >0.7 were assigned to the same cluster. 135 virtual hits were ultimately selected as representative of the various clusters (scaffolds). They were once again visually inspected for shape compatibility within the IMPα-binding site, maintenance of interactions with key binding site residues, and good alignment with the NLS in the crystal structure. 84 compounds were ultimately purchased based on availability, and validated in bioassays as described below.

### Validation of compound inhibition of IMPα/β1:C interaction using an AlphaScreen binding assay

**Figure 4:**
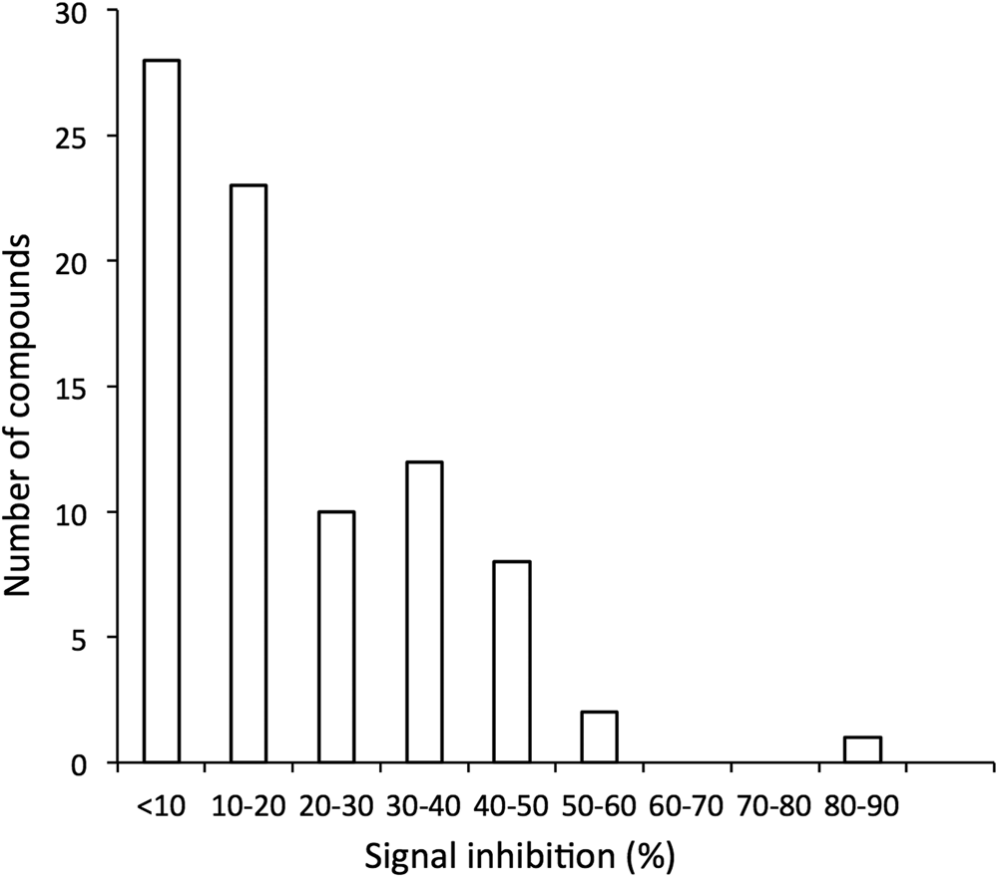
Compounds identified by *in silico* screening inhibit IMPα/β1:C binding. Pre-dimerized recombinant IMPα/β1 heterodimer (15 nM) and capsid (30 nM) were incubated together in the presence of 10 µM of each compound, and protein binding measured by AlphaScreen assay as described in Materials and Methods. The percentage inhibition of binding effected by each compound above background was standardized to DMSO-only positive control wells. Histogram shows the distribution of activity of all 84 compounds tested.

AlphaScreen protein-protein interaction assays have previously been employed to identify and confirm active compounds in high throughput screens^15,18,20,31,32^. Here we employed the assay to validate the activity of the lead compounds identified in this *in silico* screen with respect to the IMPα/β1 heterodimer, the nuclear transport active form of IMPα. Briefly, 30 nM His-tagged C was added to 15 nM pre-dimerized IMPα/β1 in the presence of 10 µM of each test compound. The signal above background was standardized to the DMSO-only control to determine the relative percent binding inhibition. Only 28 of the 84 compounds tested did not significantly (<10%) inhibit IMPα/β1:C binding, while 23 (67% of the compounds identified from the screen) inhibited the signal by >30% (Fig. 4).

**Figure 5:**
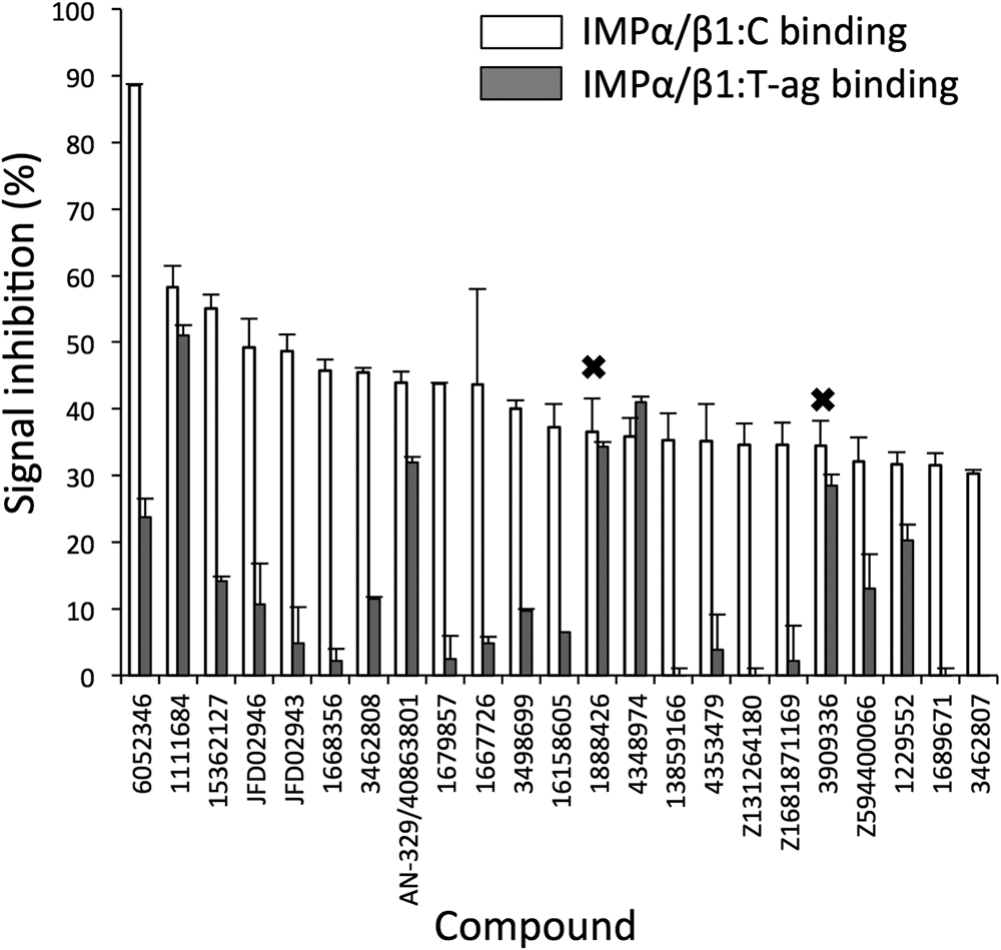
Compounds identified by *in silico* screening show specific inhibition of IMPα/β1:C binding. Compounds that inhibited the IMPα/β1:C binding signal by more than 30% were tested for their ability to inhibit IMPα/β1 (15 nM) binding to another IMPα/β1 cargo, SV40 large T-antigen (T-ag 30 nM) via AlphaScreen. Inhibition was adjusted for background and standardised to DMSO-only positive wells as per Materials and Methods. Data represent the mean ± SD (n ≥ 2). Crosses denote compounds with assay inhibition.

**Figure 6:**
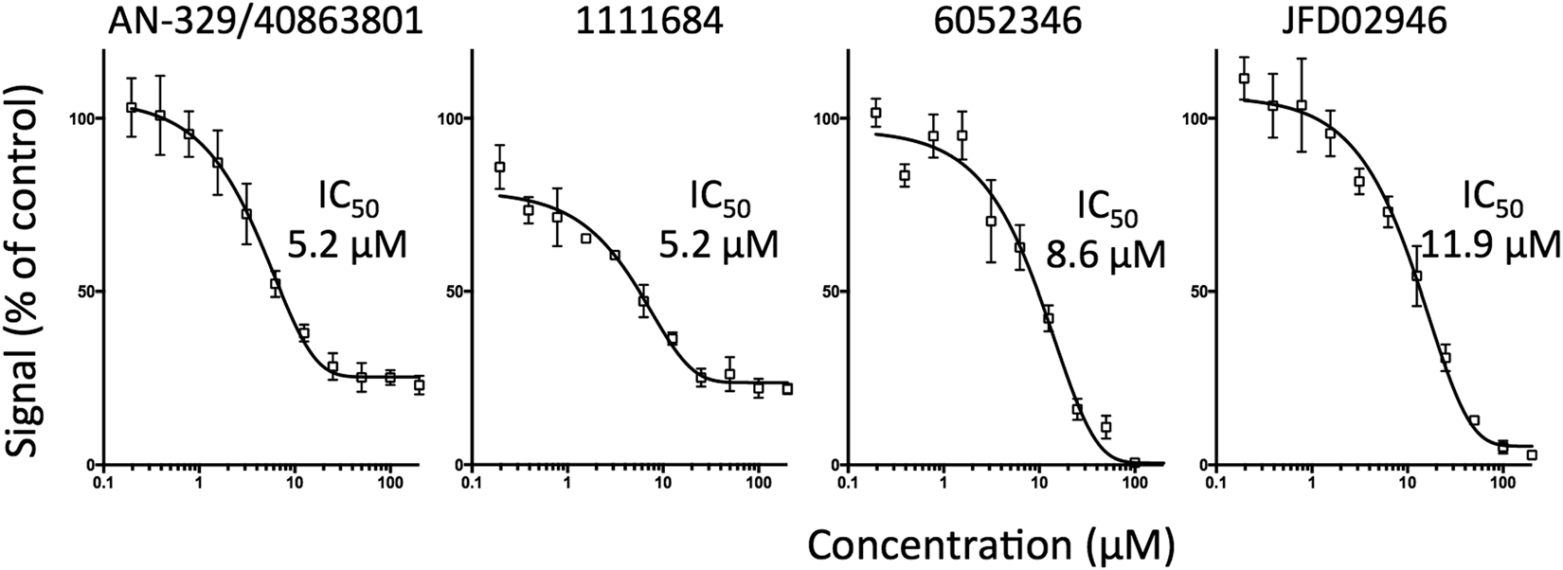
Lead compounds inhibit IMPα/β1:C binding with high affinity. 15 nM IMPα/β1 was incubated with 30 nM capsid and increasing concentrations of the individual compounds, using AlphaScreen. Values were adjusted for background and standardised to DMSO-only positive control as per Materials and Methods. Data represent the mean ± SEM (n = 3). Single, representative curves are shown from a series of two similar experiments. ND, not able to be determined due to low levels of inhibition.

Compounds that inhibited IMPα/β1:C binding >30% were counter-screened for their ability to inhibit the interaction of IMPα/β1 with an alternate cargo, a GFP fusion protein encoding the optimised NLS from Simian Virus SV40 large tumour-antigen (T-ag) (Fig. 5), to differentiate compounds whose activity was specific to the IMPα/β1:C interaction as opposed to targeting IMPα/β1 in general^33–35^, where compounds with specificity ratios (ratio of the extent of inhibition of IMPα/β1:C binding compared to that for inhibition IMPα/β1:T-ag binding) of > or < 2 were considered “specific” or “non-specific”, respectively. 17 of the 23 compounds showed specificity ratios >2, whereas 6 showed specificity ratios <2; in the case of two of these (denoted by crosses in Fig. 5), inhibition was established to be through direct inhibition of the AlphaScreen signal in control experiments using a His_6_-GST peptide (see Materials and Methods), and hence attributable to non-specific interference with the AlphaScreen assay readout. Compounds not inhibiting the IMPα/β1:T-ag interaction were deemed to be specific inhibitors of the IMPα/β1:C interaction. The seven most active specific compounds together with the two most potent non-specific compounds likely targeting IMPα/β1 were selected for IC_50_ analysis via AlphaScreen (Table 2). Of these, AN-329/40863801, 1111684, 6052346, and JFD02946, were the most active (IC_50_s < 12 µM - Fig. 6), with the two latter compounds likely to be specific inhibitors (specificity ratios > 3).

**Table 2:** Summary of structure and properties of lead compounds identified in *in silico* screening.

Structure	Compound	IC_50_ ± SD (µM)	Specificity Ratio	CC_50_ ± SD (µM)	EC_50_ ± SD (µM)	SI
	AN-329/40863801	5.2 ± 1.4	1.4	>100	>50	NA
	1111684	5.2 ± 0.6	1.1	36.4 ± 1.5	9.9 ± 1.1	3.7
	6052346	8.6 ± 1.6	3.4	>100	>50	NA
	JFD02946	11.9 ± 1.1	4.6	>100	>50	NA
	JFD02943	21.9 ± 3.5	10.2	>100	>50	NA
	1668356	22.1 ± 4.1	21.3	—	—	—
	AK-968/15362127	32.0 ± 4.6	3.9	—	—	—
	1679857	34.8 ± 1.1	17.3	—	—	—
	3462808	36.2 ± 2.3	4.0	—	—	—

IC_50_ values were determined as per Fig. 6; CC_50_ (concentration yielding 50% cytotoxicity) and EC50 (concentration yielding 50% inhibition of viral infection) were determined as per Fig. 10. Specificity ratio is the ratio of inhibition of IMPα/β1:C binding compared to inhibition of IMPα/β1:T-ag binding (AlphaScreen results as per Fig. 6). SI: Selective index, calculated as CC_50_/EC_50_.

### Inhibitors of IMPα/β1:C binding reduce C nuclear accumulation

**Figure 7:**
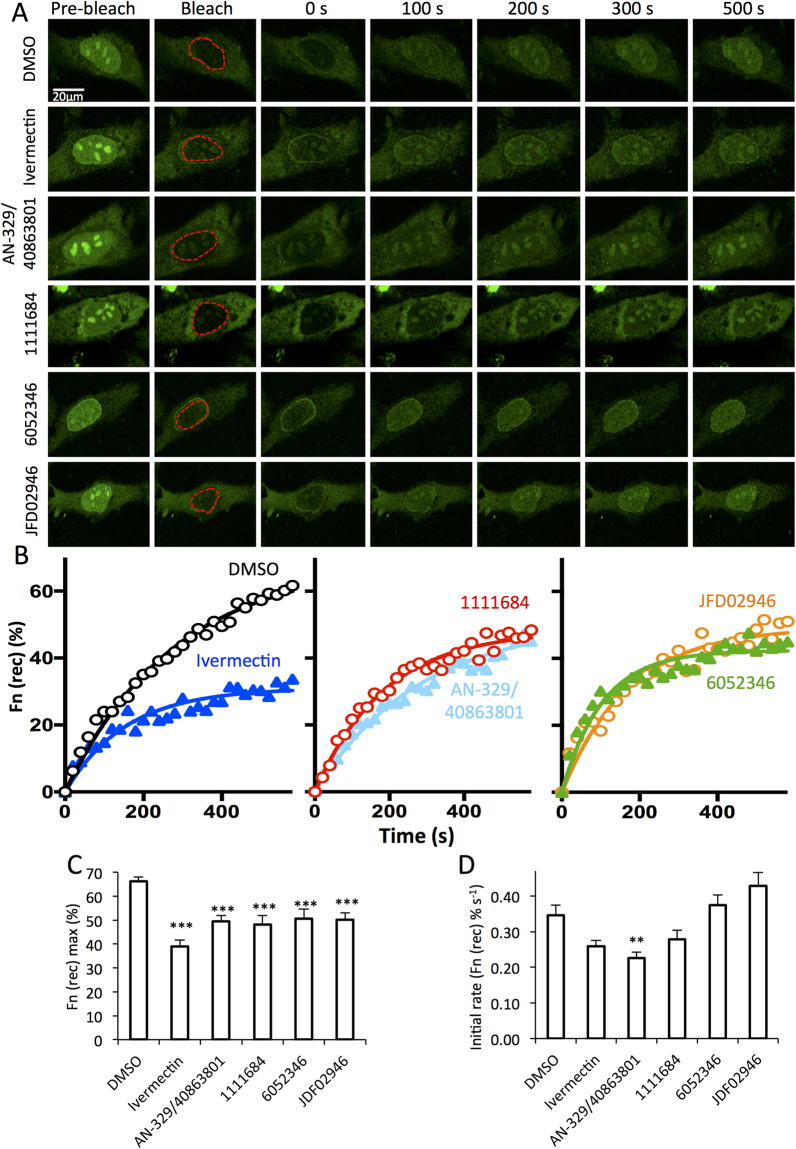
Lead compounds inhibit C nuclear import in live cells. HeLa cells transfected to express C-GFPwere treated with 50 µM compound or DMSO for 2 h as indicated, prior to FRAP analysis as per Materials and Methods. The nuclear region (outlined in red) was photo-bleached and fluorescence recovery in this region was measured every 20 s for 580 s. (**A**) Representative CLSM images of cells pre bleach, immediately following bleaching (0 s), and at the indicated times during recovery. B. Images such as those shown in A were analysed for the recovery of nuclear fluorescence (Fn (rec)). Pooled analysis of curves such as those shown in B were analysed to determine the maximal fractional recovery Fn(rec) max (**C**) and the initial rate of recovery (Fn(rec) % s^−1^ (**D**). Data represent the mean ± SEM (n ≥ 27). *p < 0.05; **p < 0.01; ***p < 0.001.

Inhibition of IMPα/β1:C binding is likely to reduce C nuclear localisation, and so the four most active compounds were tested for the ability to enter cells and inhibit C nuclear accumulation in the whole cell context^16,19^. HeLa cells were transfected to express a C-GFP fusion protein and imaged live 24 h later using confocal laser scanning microscopy (CLSM). 2 h prior to imaging, fresh media containing DMSO with or without 50 µM compound was added; the IMPα targeting inhibitor ivermectin^18,20^ was included as a positive control. Fluorescence recovery after photobleaching (FRAP) analysis was then performed to assess nuclear import kinetics; briefly, the nuclear region was irreversibly bleached using the laser at high power (bleached region indicated by the dashed red lines in Fig. 7A), and the cells subsequently imaged every 20 s for almost 10 min at lower laser power to monitor the recovery of nuclear fluorescence (Fn(rec)) (Fig. 7A,B); an indication of transport from the cytoplasm to the nucleus^36–38^. AN-329/40863801, 1111684, 6052346, and JFD02946 all significantly (p < 0.001) reduced the maximal recovery of nuclear fluorescence (Fn(rec) max; almost 30% lower than in DMSO alone treated cells - Fig. 7B,C). The general inhibitor AN-329/40863801 also significantly reduced the initial rate of C nuclear import (Fig. 7D). The results are consistent with the idea that AN-329/40863801, 1111684, 6052346, and JFD02946 are all capable of entering cells to some extent to inhibit C nuclear import, and likely act by inhibiting IMPα/β1 interaction with C, rather than slowing C import through the nuclear pore. That the levels of inhibition are not higher is likely due, at least in part, to factors such as the efficiency of compound entry into cells, and stability in medium and inside cells (see below).

### Inhibitor Selectivity

**Figure 8:**
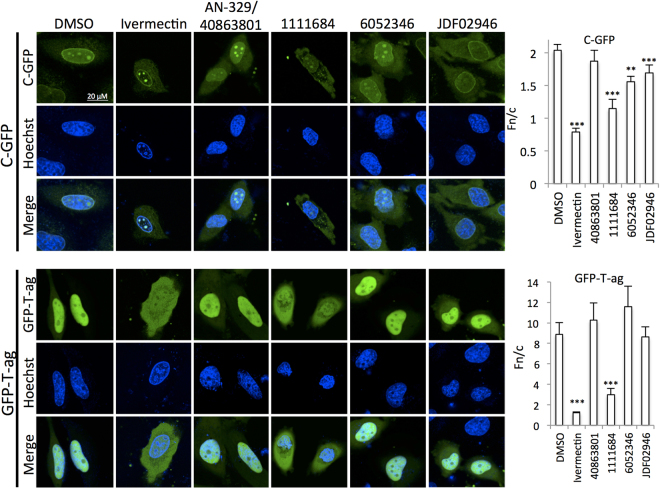
Lead compounds show specificity for Impα/β1:C. HeLa cells transfected to express C-GFP or T-ag-GFP fusion proteins were treated with 50 µM compound or DMSO as indicated for 20 h prior to CLSM imaging. Image analysis was then used to quantify the extent of nuclear accumulation in terms of the Fn/c ratio (see Materials and Methods). Data represent the mean ± SEM (n ≥ 30). *p < 0.05; **p < 0.01; ***p < 0.001.

The four most active compounds were tested along with Ivermectin for specificity in terms of their ability to inhibit nuclear accumulation of a control molecule (GFP-T-ag, containing the T-ag NLS) in transfected HeLa cells. Cells expressing either C-GFP or GFP-T-ag were treated with or without the compounds for 20 h before DAPI staining and imaging using CLSM (Fig. 8). As expected, Ivermectin and the general inhibitor 1111684 reduced nuclear accumulation of both constructs, but AN-329-40863801 had no significant effect; since anti- C transport activity was observed in the FRAP experiments (Fig. 7), the longer time frame of this experiments may be indicative of relatively low stability in culture medium/ cells. Strikingly, both the specific inhibitors 6052346 and JDF02946 showed a clear differentiation in terms of reducing C nuclear accumulation significantly (p < 0.01), but did not affect GFP-T-ag localization. Clearly, our screening approach can identify both general and selective inhibitors, the latter reducing nuclear localization of C, but not control nuclear localizing proteins such as T-ag.

### Molecular docking-based analysis of the predicted IMPα targeting compounds

**Figure 9:**
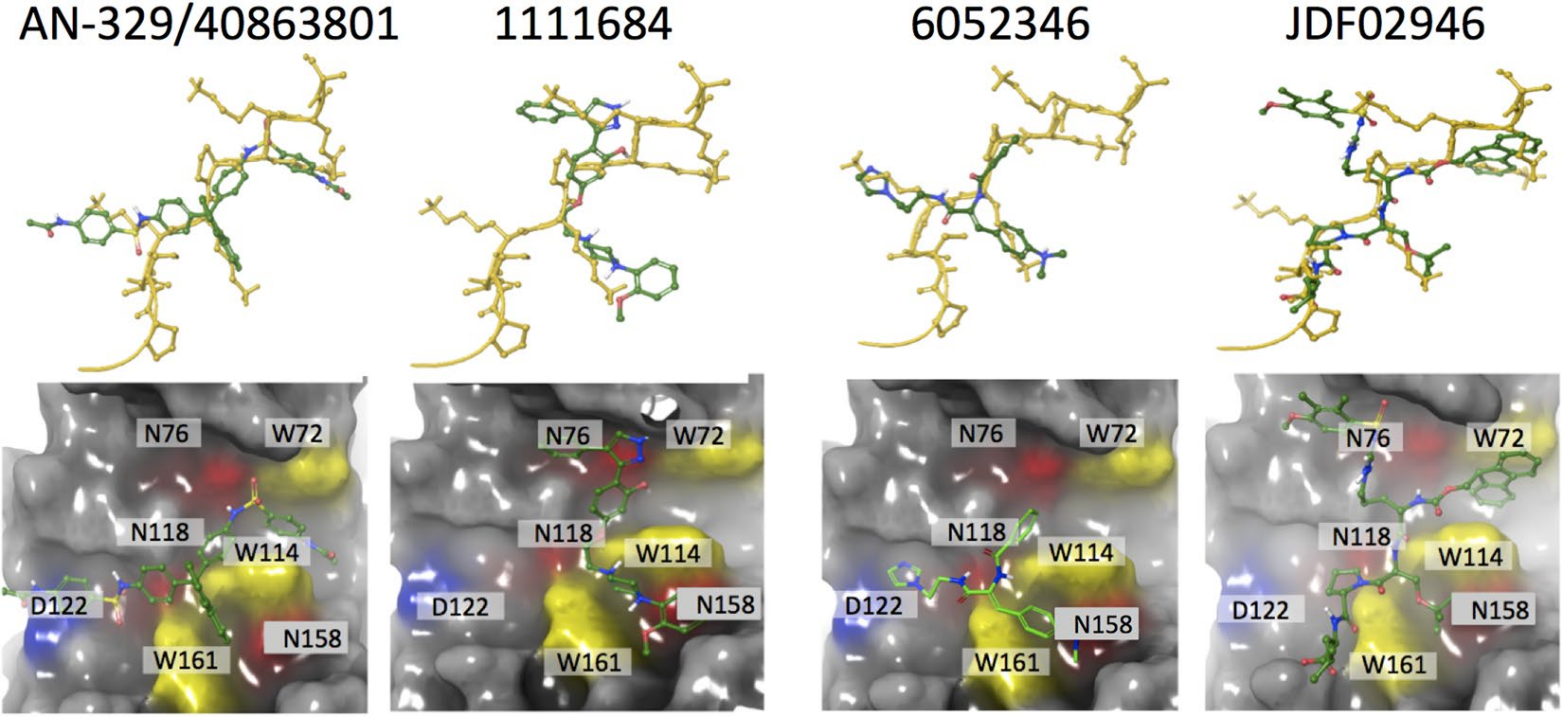
Docking of the four lead compounds to IMPα. Top: lead compounds (green) superimposed on the Core-NLS (yellow, conformation from PDB 3VE6). Bottom: IMPα binding pocket (grey) interactions with the compounds. Key residues highlighted are W72, W114 and W161, which participate in hydrophobic interactions (yellow); D122, which participates in salt bridge interactions (blue) and N76, N118 and N158, which participate in hydrogen bonding (red).

Active compounds AN-329/40863801, 1111684, 6052346 and JFD02946 represent novel classes of molecular scaffolds, and hence are of interest for molecular docking-based analysis with IMPα (see Fig. 9; Table 3). Compound AN-329/40863801 (Glide score −8.982), which inhibited IMPα/β1:C binding with an IC_50_ of 5.1 μM (specificity ratio of 1.4 – see Table 2), is inferred to be a non-specific inhibitor. Docking analysis indicated binding to IMPα predominantly through π-π stacking interactions between its aromatic rings and W72 and W114 (Fig. 9; Table 3). It also appears to use H-bonding with W72, N76 and Q111 (see Figs 2B and 9; Table 3). All of these interactions were predicted to be important for binding in our *in silico* alanine mutagenesis analysis (Fig. 2). Analogously, the other non-specific inhibitor 1111684 (Glide score −8.022), which inhibited IMPα/β1:C binding with an IC_50_ of 5.2 μM (specificity ratio of 1.1 – Table 2), interacted with the IMPα binding pocket through H-bonding with N76, S35 and W114, in addition to cation-π interactions with W114 & W161 (Fig. 9).

**Table 3:** Summary of results from molecular docking of lead compounds identified in *in silico* screening.

Residue	Interaction	AN-329/40863801	1111684	6052346	JDF02946	Min-NLS
W72	π-π	x			x	
	cation-π					x
	H-bonding	x				x
W114	π-π	x		x	x	
	cation-π		x	x		x
	H-bonding		x		x	x
W161	π-π			x		
	cation-π		x	x		
	H-bonding				x	
N76	H-bonding	x	x			x
Q111	H-bonding	x				x
Unique	H-bonding		S35	N158	R168	N118, T85, D122, G80
	Salt bridge					D200

Each of the indicated hit compounds were analyzed for interactions within the IMPα binding pocket using Ligand interaction tool (Schrodinger).

The specific inhibitor of IMPα/β1:C binding (IC_50_ of 8.6 μM and specificity ratio of 3.4 – see Table 2) 6052346 (Glide score −8.227), was found to use π-π stacking and cation-π interactions with W161 and W114 in the IMPα binding pocket, as well as H-bonding with N158 (Fig. 9; Table 3). Finally, the other specific inhibitor JDF02946 (Glide score −7.675), with an IC_50_ of 11.9 µM and specificity ratio of 4.6, bound using π-π stacking interactions with W72 and W114 as well as H-bonding with W114, W161 and R168 (Fig. 9; Table 3).

Thus, all four compounds, both specific and non-specific, as well as the Min-NLS peptide, (Table 3) interact with one or more of W72, W161, or W114 in the NLS binding pocket, through cation-π, π-π stacking hydrophobic or H-bonding interactions, or a combination thereof. Interestingly, π-π stacking interactions were used by all of the compounds, but not by the Min-NLS itself (Table 3). The apparent specificity of 6052346 and JDF02946 would appear to be a result of unique interactions that are distinct to those of the two non-specific binders (AN-329/40863801 and 1111684) which, apart from targeting the W residues, as indicated, also bind to residues at the “top” of the binding pocket (N76 and Q111), which are in close proximity and interact with K at position #9 of the Min-NLS (Fig. 2B). Binding with N76 is not seen for 6052346 and JDF02946. These interact, in contrast to the general inhibitors, with residues at the bottom of the binding pocket (N158 and R168), which are in close proximity to and interact with K at positions #6 and 7 of the Min-NLS (see also Fig. 2B). They also bind W161 using π-π stacking and H-bond interactions that are not seen for either the Min-NLS or the two non-specific compounds.

### Lead compound 1111684 reduces C nuclear localization in infected cells and inhibits viral replication

**Figure 10:**
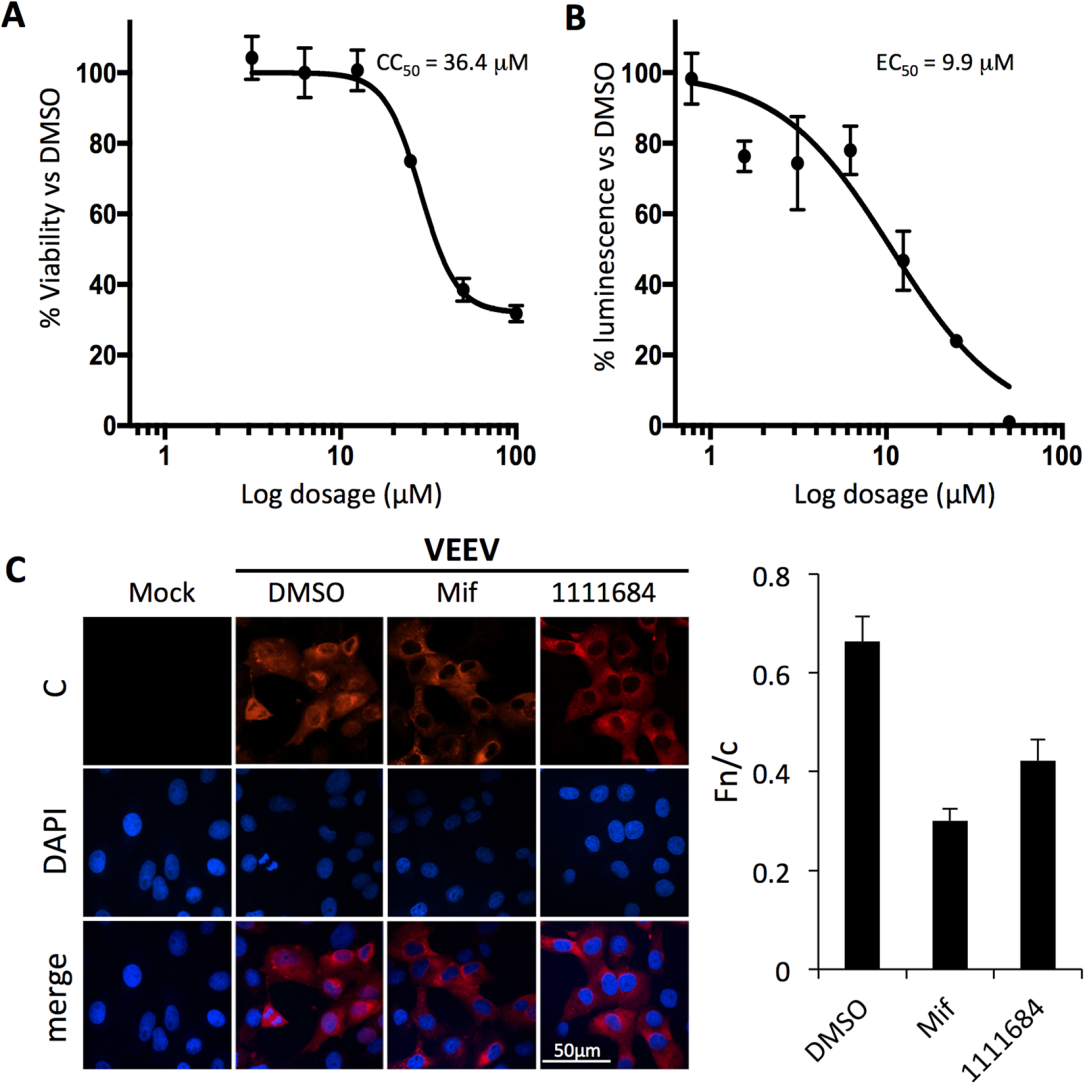
Compound 1111684 alters VEEV capsid distribution and inhibits viral replication. (**A**) Vero cells were treated with serial dilutions of 1111684 in the vehicle DMSO. Luminescence was measured using the Promega CellGlo Viability Assay (see Materials and Methods) at 24 h post-treatment. Data represent the mean ± SD (n = 4) luminescence as a percentage normalized to that of DMSO-treated cells. (**B**) Vero cells were pretreated with DMSO or increasing concentrations of 1111684 2 h prior to infection, and then infected with VEEV-TC83luc at a multiplicity of infection (MOI) of 1 in the continued presence of 1111684. The BrightGlo Luciferase Assay was performed at 16 h post-infection (p.i.). Data represent the mean ± SD (n = 4) luminescence as a percentage normalized to that of DMSO-treated cells. (**C**) Vero cells were pretreated with DMSO (0.1%) with or without Mifepristone or 1111684 (10 μM) for 2 h prior to infection with VEEV-TC83 at an MOI of 0.1 in the continued presence of the respective inhibitors. Mock-infected cells were untreated and uninfected. At 16 h p.i., cells were fixed and probed for C (red) and DAPI stained (blue). Nuclear to cytoplasmic fluorescence ratios (Fn/c) were determined as previously^18,20^ (right). **p < 0.005; ***p < 0.0001.

The four hit compounds with the lowest IC_50_ values were analysed for their potential cytotoxicity, with all displaying CC_50_ values > 100 µM (Table 2), with the exception of 1111684 (CC_50_ of 36.4 uM – see Fig. 10A). A reporter virus encoding firefly luciferase (VEEV-luc) was then used to test whether the compounds could inhibit viral replication, with only 1111684 showing a marked inhibitory effect on VEEV infection (EC_50_ of 9.9 µM – see Fig. 10B). That the other compounds did not show marked antiviral activity is likely because of one or more limiting factors to *in vivo* efficacy, such as poor stability in culture medium/cells (see above), and/or low efficiency of entering cells. The selectivity index (CC_50_/EC_50_) of 1111684 was 3.7, but it should be noted that while maximal toxicity was less than 70%, 1111684 could achieve close to 100% suppression of VEEV replication. To confirm that the effects on viral replication were due to interference with the IMPα/β1:C interaction, C localization was examined in the infected cells by CLSM. VEEV C was observed in both nuclear and cytoplasmic compartments in the presence or absence of DMSO (Fig. 10C). Treatment with either Mifepristone or 1111684 resulted in reduced nuclear C staining, concomitant with increased cytoplasmic staining (Fig. 10C), confirmed by quantitation of the nuclear to cytoplasmic fluorescence ratio (Fn/c - Fig. 10C; histogram right). Collectively, the results show that 1111684, in similar fashion to Ivermectin and Mifepristone, inhibits VEEV replication, at least in part through reducing C nuclear accumulation.

### Anti-VEEV activity of lead compound 1111684 is through targeting the IMPα/β1:C interaction

**Figure 11:**
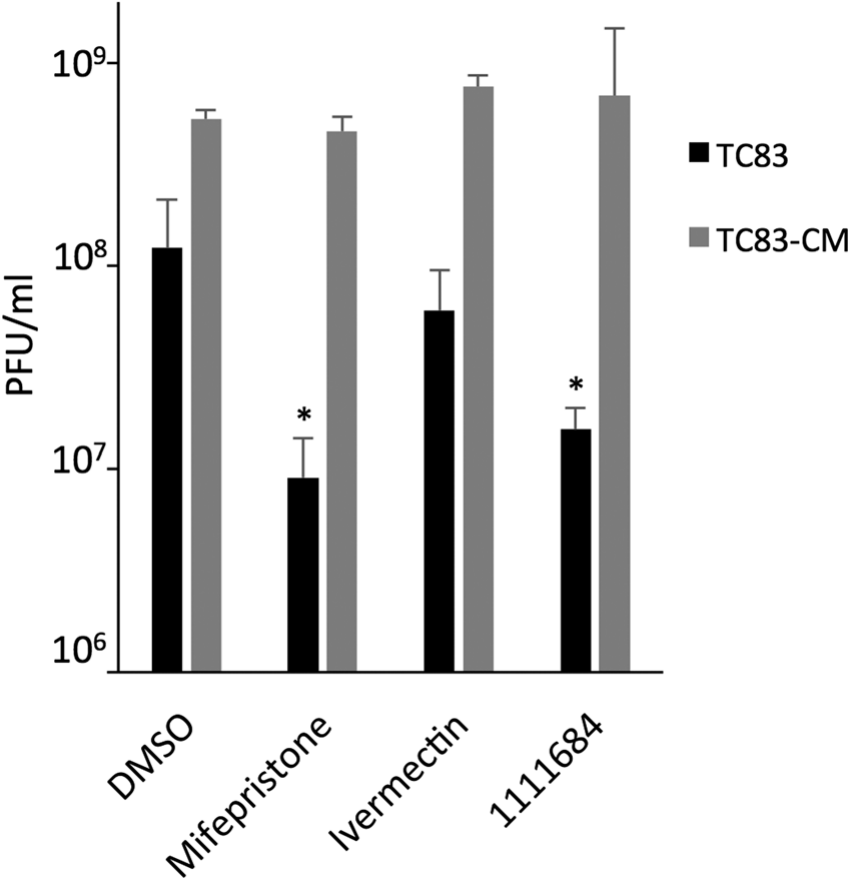
Antiviral activity of 1111684 is through targeting the IMPα/β1:C interaction. Vero cells were pretreated for 2 h with Mifepristone (10 μM), Ivermectin (1 μM), 1111684 (10 μM), or a DMSO only control. Supernatants were removed, and virus added (WT or CM, MOI 0.1). After 1 h, the medium was replaced with medium containing compound. After 16 h, supernatants were collected and plaque assays performed to determine viral titers (using Vero or BHK.J cells for TC83-WT or TC83-CM virus, respectively). Data represent mean ± SD (n = 3). *p < 0.05 compared to DMSO.

TC83-CM is a VEEV mutant encoding C containing a mutated NLS^17,39^ that is unable to bind IMPα, and hence is not imported into the nucleus^17,19^; as a result, the virus is attenuated *in vivo* and fails to induce cytopathic effects in cultured cell systems^39^. We hypothesized that if 1111684’s mode of action is to interfere with C’s ability to disrupt cellular processes through binding IMPα, TC83-CM replication should not be sensitive to 1111684. Vero cells were accordingly pretreated with 1111684, Mifepristone or Ivermectin as controls, or DMSO prior to infection with wild-type TC83 (TC83-WT) or TC83-CM, after which the media containing the drug treatment was replaced. Supernatants were collected 16 hpi and plaque assays performed. Although TC83-CM, like many attenuated alphaviruses, fails to form plaques in Vero cells, it is able to do so in BHK-21 cells^12,39,40^, enabling us to perform assessment of infectious virus production in BHK-21 cells^39^. Treatment with either 10 µM 1111684 or Mifepristone significantly reduced the titer of wild-type TC83 compared to DMSO treatment, while 1 µM Ivermectin reduced the titer to a lesser extent. Conversely, none of the treatments affected TC83-CM titers (Fig. 11), consistent with the idea that the antiviral mode of action of 1111684, Ivermectin, and Mifepristone is through targeting the IMPα/β1:C NLS interaction. The fact that 1111684 shows robust anti-VEEV activity comparable to that of Ivermectin makes it an interesting prospect for further development.

## Discussion

This is the first study to use the solved structure of IMPα bound to VEEV-C NLS as the basis for SBDD to identify small molecule inhibitors of the IMPα:C interaction. A key to our approach was to define and use the minimal/core NLS of C (positions #6-KKPK-#9) to delineate the C NLS binding pocket on IMPα, which we investigated in more detail using *in silico* alanine mutagenesis/docking analysis to identify the likely key residues of IMPα involved in binding. Since small molecules, unlike the C NLS, cannot span the length and width of the binding pocket, we incorporated novel hydrophobic interactions, as well as those used by the Min-NLS (π-π interactions, eg. between the molecule aromatic rings and the indoles of W161, W114 and W72, and cation- π interactions, respectively) to increase the likelihood of finding high affinity inhibitors. This strategy was clearly a key to our success in identifying compounds of interest.

Based on the findings here, it seems reasonable to postulate that the residues of IMPα that interact with the K residues at positions #6 and #7 of the VEEV C NLS (residues highlighted in green in Fig. 2B) could be key to improving binding/identifying more potent and specific inhibitors either in future screens or as part of an SAR effort. Our *in silico* alanine scanning analysis (Fig. 2A) suggested that interactions with IMPα residues D122 > N118 > D200 > T85 > N158 (in descending order of importance) should be taken into account. Our specific binders identified appear to interact with N158, R168 and W161, but not D122, which showed the biggest energy change upon substitution with A (and is clearly involved in IMPα interaction with NLSs for other viral proteins; eg. Tay *et al*.^27^). All of the results here give important new information on the NLS-binding site of IMPα, and provide the basis to refine and improve the present screen. Further, our results highlight the potential to achieve general or specific inhibition of binding interactions as required by targeting key residues for disruption. This broadens the potential pool of antiviral targets, and may allow current inhibitors to be modified to increase specificity and minimize toxicity due to off-target effects.

Our lead compound, 1111684, significantly inhibited replication of TC83-WT but not TC83-CM, strongly implying that it targets the IMPα/β1:C NLS interaction, which is abrogated in TC83-CM because of the inactivating mutations in the C NLS^17,40^. The fact that TC83-CM shows strong attenuation *in vivo* ^39^ underlines the importance of C nuclear entry to VEEV pathogenesis. The fact that TC83-CM is able to replicate in Vero/BHK cells to comparable extents to TC83-WT in the absence of 1111684 (see Fig. 11), is consistent with the idea that C nuclear entry is not essential for replication *in vitro* ^40^, the basis of which, at this stage, is not clear. Excitingly, what our results do highlight is the potential that 1111684 may have in treating VEEV infection *in vivo*.

In conclusion, this is the first time that *in silico* approaches have been used successfully to target IMPα’s NLS-binding site, with SAR highly likely to improve the potency and specificity of our lead IMPα inhibitor 1111684 that inhibits VEEV in the µM range. These results indicate that targeting the IMPα NLS-binding site with relatively small molecules, though challenging, is feasible, and has great potential to generate potent antiviral agents of therapeutic utility in the future.

## Methods

### Preparation of the IMPα structure for docking

Virtual screening was carried out on the IMPα:VEEV NLS crystal structure [PDB:3VE6, 2.83 Å resolution]. To prepare the protein structure for docking, all solvent molecules were deleted and bond orders for the ligand and the protein were adjusted. The missing H-atoms were added, and side chains then energy-minimised using the OPLS-2005 force field (Maestro software Schrodinger). The ligand-binding site was defined using Receptor Grid generation (Schrodinger) centred on the Min-NLS within PDB 3VE6; default settings were used for all other parameters.

### Ligand preparation and virtual screening

A database of 1.5 M publically available compounds from established suppliers ASINEX (Moscow, Russia), Otava (Vaughan, Ontario, Canada), Specs (Zoetermeer, Netherlands), Enamine (Kyiv, Ukraine), and Thermofisher (Maybridge) (Scoresby, Victoria, Australia) was compiled and prepared using Ligprep (Schrodinger), which added H-atoms, generated ionization and tautomer states and eventually converted to low energy 3D structure with correct chiralities. Minimization was performed using OPLS_2005 force field and Epik ionizer at the standard pH of 5 to 9 and with a maximum of 32 stereoisomers per structure. Confirmatory assays were performed using compounds purchased from the relevant companies as required; compounds were dissolved in DMSO for use.

### Clustering

To cluster compounds based on structural similarity, Canvas (Schrodinger) was used, employing the multiple level atom neighborhoods method^30^. Compounds with Tanimoto cutoff values > 0.7 were assigned to the same cluster.

### Protein expression constructs

The protein coding sequence for VEEV C was PCR-amplified using plasmid pTC83 as a template (bp7563-8387) and inserted upstream of a Gly_4_ linker region and eGFP sequence using an overlap PCR approach. This fragment was then inserted into pcDNA3.1 using restriction enzymes BamHI and NotI, cleavage sites for which were included in the 5′ and 3′ primer sequences respectively to create pcDNA3.1_VEEVC-GFP for transfection experiments. Plasmid pcDNA3.1_VEEVC-GFP was then used as a template for PCR amplification of the C coding sequence using primers to incorporate an in-frame His_6_ tag in the 5′ coding sequence, as well as flanking NdeI and HindIII restriction sites. This fragment was then inserted into plasmid vector pCOLDIV (Takara Bio, Japan) to create pColdIV-C for bacterial expression. The GFP-T-ag NLS containing fusion protein, encoded by the plasmid vector pDEST53-T-ag(111–135) for mammalian cell expression has been described^18,20^.

### Protein expression in bacteria

NiCO21 (DE3) cells carrying plasmid pColdIV-C were grown overnight in Overnight Express TB (Merck, Germany) at 28 °C, followed by a further 24 h at 20 °C. Cells were harvested by centrifugation at 3500 rpm, resuspended in 100 mM Na_2_HPO_4_, 150 mM NaCl, pH 7.4, and immediately frozen at −20 °C. Upon thaw, 1 ml of popculture lysis solution (Merck), 1 protease inhibitor cocktail tablet and 10 µg/ml DNase I was added and the cells sonicated at 10 hz for 6 × 30 s cycles of sonication followed by 30 s of cooling on ice. Lysates were cleared by centrifugation (16,000 rpm, 4 °C) and then incubated with Ni-NTA agarose resin (Qiagen, Germany) for 3 h at 4 °C on a rotator. The resin was washed with 30x resin volume in 100 mM Na_2_HPO_4_, 300 mM NaCl, pH 7.4, and eluted in 100 mM Na_2_HPO_4_, 150 mM NaCl, 500 mM imidazole, pH 7.4. The eluate was further purified by gel filtration using a S200 column (100 mM Na_2_HPO_4_, 150 mM NaCl, pH 7.4). Enriched fractions were pooled and concentrated using an Amicon centrifuge filter device with a 10 kDa MWCO (Merck Millipore, USA). IMPα2 and IMPβ1 were expressed as GST-tagged proteins in bl21(pREP4) *E. coli* cells and purified under native conditions as previously^34,35^. Prior to use, IMPβ1 and IMPα2 were dimerized at a 1:1 ratio in intracellular buffer (110 mM KCl, 5 mM NaHCO_3_, 5 mM MgCl_2_, 1 mM EGTA, 0.1 mM CaCl_2,_ 20 mM HEPES, pH 7.4) at a concentration of 13.6 µM for 15 min at room temperature^41^. The optimised T-ag NLS-GFP fusion protein was purified as a His_6_-tagged peptide under denaturing conditions from bl21(pREP4) as previously^32^. His_6_-GST was from Abcam (UK). Protein concentrations were estimated by SDS-PAGE and Coomassie staining, and/or quantified by Bradford Protein Assay (Bio-Rad Laboratories, USA).

### AlphaScreen assay

AlphaScreen binding assays were performed as previously in triplicate in white opaque 384 well plates in a final volume of 25 µl^20,32^. Briefly, 5 µl of PBS (137 mM NaCl, 2.7 mM KCl, 10 mM Na_2_HPO_4_, 1.8 mM KH_2_PO_4_, pH 7.4) was added to each well, followed by 5 µl of either C or T-ag NLS-GFP (final concentration, 30 nM), along with 5 µl of compound in 0.1% DMSO, diluted to a final concentration of 10 µM in PBS. 5 µl of pre-dimerised IMPα/β1 was added (final concentration of 15 nM) followed by 30 min incubation at room temperature. Finally, 5 µl AlphaScreen bead mix (1/250 dilution of nickel NTA donor beads, 1/250 dilution of GST acceptor beads, and 0.1% BSA in PBS) was added and the plates incubated in the dark at room temperature for 16 h. Plates were read on an Enspire plate reader (PerkinElmer) the following morning. To assess if compounds were inhibiting the AlphaScreen reaction directly (false positives), a His_6_-GST fusion protein, which binds both beads simultaneously, generating a robust signal was used. The AlphaScreen assay was performed as above except that His_6_-GST was substituted for IMPα/β1 and C at a final concentration of 1.25 nM with the volume made up to 25 μl with PBS. DMSO alone positive controls were included on each assay plate, along with negative controls in which one of the proteins was removed. The signal from the negative control wells was subtracted from all samples, including the positive control, and the % inhibition determined in relation to the positive control. IC_50_ values were determined from AlphaScreen binding assays essentially as above, but with binding assessed in the presence of a range of compound concentrations (0.2 µM to 200 µM). Data was analysed using Prism software (GraphPad Software, Inc.) and IC_50_s determined by fitting one-phase dissociation curves to the data.

### Fluorescence imaging/fluorescence recovery after photobleaching (FRAP)

HeLa cells were maintained in Eagle’s Minimum Essential Medium (EMEM) supplemented with 10% fetal calf serum (FCS), 1 x GlutaMAX (Thermofisher, USA), and 1 x non-essential amino acids (NEA,Thermofisher) at 37 °C with 5% CO_2_. Cells grown on glass coverslips were transfected with 1 µg of either plasmid pcDNA3.1_VEEVC-GFP (encoding C-GFP fusion protein) or plasmid pGFP-T-ag using lipofectamine 3000 (Invitrogen) as per the manufacturer’s instructions.

For analysis of C or control protein localization at steady state, HeLa cells were transfected to express either the C-GFP or GFP-T-ag fusion proteins. 6 h post-transfection, media was replaced with that containing 50 µM compound, and cells were Hoechst stained and imaged 20 h later by CLSM. Digitised images were analysed using imageJ to measure the Fn/c ratio using the formula; Fn/c = (Fn − Fb)/(Fc − Fb), where Fn is the nuclear fluorescence, Fc, the cytoplasmic fluorescence, and Fb the background fluorescence.

For FRAP experiments, culture medium was replaced with medium containing DMSO ± 50 µM compound and the cells incubated for 2 h at 37 °C, prior to imaging by confocal laser scanning microscopy (CLSM) using an Olympus FV1000 microscope equipped with temperature control and a 100x oil immersion lens. FRAP analysis was performed as previously^36–38^, whereby nuclei were selectively bleached (12 scans at 100% laser power, 488 nm laser) and recovery monitored every 20 s for 580 s. Digitised images were analysed using ImageJ software (National Institute of Health, Bethesda, MD) to determine the % recovery of nuclear fluorescence (Fn(rec)), calculated using the formula; Fn(rec)t_x_ = [Fn(t_x_) − Fn(t_0_)]/[Fn(pre) − Fn(t_0_)] ∗ 100, where Fn(t_x_) is the nuclear fluorescence minus the background fluorescence at x seconds post-bleach, and Fn(pre) is the nuclear fluorescence minus background fluorescence pre-bleach. One-phase association curves were fitted using Prism to calculate the Fn(rec) max. Statistical analyses were performed using Students t-test.

### Immunofluorescence

Vero cells grown on coverslips in a 6-well plate were processed for immunofluorescence analysis as previously^16^. Antibodies used were anti-VEEV-C protein (BEI Resources, NR-9403) goat primary antibody (1:1000 dilution) and Alexa Fluor 568 donkey anti-goat secondary antibody (1:500 dilution). Slides were imaged using an oil-immersion 60X objective lens on a Nikon Eclipse TE 2000-U confocal microscope, with all samples subjected to four line averaging. At least three images were taken for each sample, and then processed using the Nikon NIS-Elements AR Analysis 3.2 software. Digitized images were analyzed as previously using ImageJ 1.47 public domain software^16^ (see also above).

### **CC**_**50**_**and EC**_**50**_**assays**

CC_50_s were measured in Vero cells incubated with serially diluted drugs or DMSO vehicle alone for 24 h. Cytotoxicity was measured using Promega’s CellGlo Cell Viability assay, according to the manufacturer’s specifications. EC_50_s were determined using the reporter virus, VEEV TC-83 luciferase. Serial dilutions of each compound were used to pre-treat Vero cells for 2 h prior to infection with the reporter virus. After infection, cells were washed and the drug media replaced. Inhibition of viral replication was measured 16 h post-infection using Promega’s BrightGlo Luciferase assay according to the manufacturer’s specifications.

### Plaque assays

Vero cells were cultured at 37 °C in a humidified atmosphere containing 5% CO_2_. 1111684 (10 μM), Mifepristone (10 μM), or Ivermectin (1 μM) was diluted in DMEM (supplemented with 10% FBS, 1% L-glutamine, and 1% penicillin/streptomycin), and used to pre-treat Vero cells for 2 h. Supernatants were removed, and virus diluted in supplemented DMEM was added to the cells for 1 h. Virus inoculum was removed, cells were washed once with sterile 1X PBS, and compound media replaced. Supernatants were collected at 16 h post-infection (hpi).

To determine viral titers, crystal violet plaque assays were performed^16^. Briefly, infected supernatants were serially diluted 1:10 in supplemented DMEM then added to confluent Vero cells (TC83-WT) or BHK.J (a line of BHK-21) cells (TC83-CM) as previously^39,40^. A 1:1 mixture of 2× Eagle’s Minimal Essential Medium (EMEM) (supplemented with 5% FBS, 1% minimum essential amino acids, 1% sodium pyruvate, and 2% penicillin/streptomycin) and 0.6% agarose in purified water were added 1 h later to each well. 48 h later, plates were fixed with 10% formaldehyde for at least 1 h. Once the agarose plugs were removed, cells were stained with 1% crystal violet in 20% ethanol and purified water. Plaques were counted, and triplicate samples averaged.

### Data availability

The datasets generated during and/or analysed during the current study are available from the corresponding author upon reasonable request.

## Electronic supplementary material

Supplementary Table S1

## References

[1] Wang HL, O’Rear J, Stollar V (1996). Mutagenesis of the Sindbis virus nsP1 protein: effects on methyltransferase activity and viral infectivity. Virology.

[2] Strauss JH, Strauss EG (1994). The Alphaviruses: Gene Expression, Replication, and Evolution. Microbiology and Molecular Biology Reviews.

[3] Frolov, I. Persistent infection and suppression of host response by alphaviruses. *Archives of virology. Supplementu*m, 139–147 (2004). 10.1007/978-3-7091-0572-6_12 15119769

[4] Griffin DE (2010). Emergence and re-emergence of viral diseases of the central nervous system. Prog Neurobiol.

[5] Griffin, D. E. In *Field*s’ Viro*lo*gy Vol. 1 (eds D. M. Knipe & P. M. Howley) Ch. 31, 1023–1069 (Wolters Kluwer Health/Lippincott Williams & Wilkins, 2007).

[6] Cheng RH (1995). Nucleocapsid and glycoprotein organization in an enveloped virus. Cell.

[7] Paessler, S. & Taylor, K. G. In *N*on*-Fl*avivir*us* Ence*phali*tis (eds Tkachev) (InTech, 2011).

[8] Simmons JD (2009). Venezuelan equine encephalitis virus disrupts STAT1 signaling by distinct mechanisms independent of host shutoff. J Virol.

[9] Aguilar PV (2008). Structural and nonstructural protein genome regions of eastern equine encephalitis virus are determinants of interferon sensitivity and murine virulence. J Virol.

[10] Ryman KD, Klimstra WB (2008). Host responses to alphavirus infection. Immunological Reviews.

[11] Atasheva S, Garmashova N, Frolov I, Frolova E (2008). Venezuelan equine encephalitis virus capsid protein inhibits nuclear import in Mammalian but not in mosquito cells. J Virol.

[12] Garmashova N (2007). The Old World and New World alphaviruses use different virus-specific proteins for induction of transcriptional shutoff. J Virol.

[13] Fulcher AJ, Jans DA (2011). Regulation of nucleocytoplasmic trafficking of viral proteins: an integral role in pathogenesis?. Biochim Biophys Acta.

[14] Caly L, Wagstaff KM, Jans DA (2012). Nuclear trafficking of proteins from RNA viruses: potential target for antivirals?. Antiviral Res.

[15] Fraser JE (2014). A nuclear transport inhibitor that modulates the unfolded protein response and provides *in vivo* protection against lethal dengue virus infection. J Infect Dis.

[16] Lundberg L (2013). Nuclear import and export inhibitors alter capsid protein distribution in mammalian cells and reduce Venezuelan Equine Encephalitis Virus replication. Antiviral Res.

[17] Lundberg L (2016). Selective Inhibitor of Nuclear Export (SINE) Compounds Alter New World Alphavirus Capsid Localization and Reduce Viral Replication in Mammalian Cells. PLoS Negl Trop Dis.

[18] Wagstaff KM, Sivakumaran H, Heaton SM, Harrich D, Jans DA (2012). Ivermectin is a specific inhibitor of importin alpha/beta-mediated nuclear import able to inhibit replication of HIV-1 and dengue virus. Biochem J.

[19] Atasheva S, Fish A, Fornerod M, Frolova EI (2010). Venezuelan equine Encephalitis virus capsid protein forms a tetrameric complex with CRM1 and importin alpha/beta that obstructs nuclear pore complex function. J Virol.

[20] Wagstaff KM, Rawlinson SM, Hearps AC, Jans DA (2011). An AlphaScreen(R)-based assay for high-throughput screening for specific inhibitors of nuclear import. J Biomol Screen.

[21] Kalid O, Toledo Warshaviak D, Shechter S, Sherman W, Shacham S (2012). Consensus Induced Fit Docking (cIFD): methodology, validation, and application to the discovery of novel Crm1 inhibitors. J Comput Aided Mol Des.

[22] Zhao L, Chmielewski J (2005). Inhibiting protein-protein interactions using designed molecules. Curr Opin Struct Biol.

[23] Pelay-Gimeno M, Glas A, Koch O, Grossmann TN (2015). Structure-Based Design of Inhibitors of Protein-Protein Interactions: Mimicking Peptide Binding Epitopes. Angew Chem Int Ed Engl.

[24] Roman N, Christie M, Swarbrick CM, Kobe B, Forwood JK (2013). Structural characterisation of the nuclear import receptor importin alpha in complex with the bipartite NLS of Prp20. PLoS One.

[25] Marfori M, Lonhienne TG, Forwood JK, Kobe B (2012). Structural basis of high-affinity nuclear localization signal interactions with importin-alpha. Traffic.

[26] Dingwall C, Robbins J, Dilworth SM, Roberts B, Richardson WD (1988). The nucleoplasmin nuclear location sequence is larger and more complex than that of SV-40 large T antigen. The Journal of Cell Biology.

[27] Tay MY (2016). The C-terminal 18 Amino Acid Region of Dengue Virus NS5 Regulates its Subcellular Localization and Contains a Conserved Arginine Residue Essential for Infectious Virus Production. PLoS Pathog.

[28] Anand P, Nagarajan D, Mukherjee S, Chandra N (2014). ABS-Scan: In silico alanine scanning mutagenesis for binding site residues in protein-ligand complex. F1000Res.

[29] Sliwoski G, Kothiwale S, Meiler J, Lowe EW (2014). Computational methods in drug discovery. Pharmacol Rev.

[30] Filimonov D, Poroikov V, Borodina Y, Gloriozova T (1999). Chemical Similarity Assessment through Multilevel Neighborhoods of Atoms:  Definition and Comparison with the Other Descriptors. Journal of Chemical Information and Modeling.

[31] Glickman JF (2002). A Comparison of ALPHAScreen, TR-FRET, and TRF as Assay Methods for FXR Nuclear Receptors. Journal of Biomolecular Screening.

[32] Wagstaff KM, Jans DA (2006). Intramolecular masking of nuclear localization signals: analysis of importin binding using a novel AlphaScreen-based method. Anal Biochem.

[33] Xiao, C.-Y., Jans, P. & Jans, D. A. Negative charge at the protein kinase CK2 site enhances recognition of the SV40 large T-antigen NLS by importin: effect of conformation. *FEBS Letters* **440**, 297-301, 0.1016/S0014-5793(98)01478-1 (1998). 10.1016/s0014-5793(98)01478-1 9872390

[34] Xiao CY, Hubner S, Jans DA (1997). SV40 Large Tumor Antigen Nuclear Import Is Regulated by the Double-stranded DNA-dependent Protein Kinase Site (Serine 120) Flanking the Nuclear Localization Sequence. Journal of Biological Chemistry.

[35] Hubner S, Xiao CY, Jans DA (1997). The Protein Kinase CK2 Site (Ser111/112) Enhances Recognition of the Simian Virus 40 Large T-antigen Nuclear Localization Sequence by Importin. Journal of Biological Chemistry.

[36] Ng IH, Bogoyevitch MA, Jans DA (2014). Cytokine-induced slowing of STAT3 nuclear import; faster basal trafficking of the STAT3beta isoform. Traffic.

[37] Roth DM, Moseley GW, Pouton CW, Jans DA (2011). Mechanism of microtubule-facilitated “fast track” nuclear import. J Biol Chem.

[38] Kuusisto HV, Wagstaff KM, Alvisi G, Roth DM, Jans DA (2012). Global enhancement of nuclear localization-dependent nuclear transport in transformed cells. FASEB J.

[39] Atasheva S, Kim DY, Frolova EI, Frolov I (2015). Venezuelan equine encephalitis virus variants lacking transcription inhibitory functions demonstrate highly attenuated phenotype. J Virol.

[40] Atasheva S, Krendelchtchikova V, Liopo A, Frolova E, Frolov I (2010). Interplay of acute and persistent infections caused by Venezuelan equine encephalitis virus encoding mutated capsid protein. J Virol.

[41] Baliga BC (2003). Role of prodomain in importin-mediated nuclear localization and activation of caspase-2. J Biol Chem.

