# Author Correction: Novel inhibitors targeting Venezuelan equine encephalitis virus capsid protein identified using *In Silico* Structure-Based-Drug-Design

**DOI:** 10.1038/s41598-018-31644-7

**Published:** 2018-09-04

**Authors:** Sharon Shechter, David R. Thomas, Lindsay Lundberg, Chelsea Pinkham, Shih-Chao Lin, Kylie M. Wagstaff, Aaron Debono, Kylene Kehn-Hall, David A. Jans

**Affiliations:** 1 Shechter Computational Solutions, Andover, MA USA; 2 Nuclear Signaling Laboratory, Department of Biochemistry and Molecular Biology School of Biomedical Sciences, Monash University, Melbourne, Australia; 3 National Center for Biodefense and Infectious Diseases, School of Systems Biology, George Mason University, Manassas, VA USA; 4 Monash Institute of Pharmaceutical Sciences, Parkville, Victoria Australia

Correction to: *Scientific Reports* 10.1038/s41598-017-17672-9, published online 18 December 2017

This Article contains errors.

The reference to the PDB entry was missing. It is included below as Reference 1,and should be cited in the Methods section under the subheading ‘Preparation of the IMPα structure for docking’ as follows:

“Virtual screening was carried out on the IMPα:VEEV NLS crystal structure [PDB:3VE6, 2.83 Å resolution]^1^.”

## References

[1] Fan, F. Crystal Structure Analysis of Venezuelan Equine Encephalitis Virus Capsid Protein NLS and Importin Alpha. doi: 10.2210/pdb3VE6/pdb.

