# Supplementary material for: Novel inhibitors targeting Venezuelan equine encephalitis virus capsid protein identified using In Silico Structure-Based-Drug-Design
Source: Sci Rep. 2017 Dec 18;7:17705. doi: 10.1038/s41598-017-17672-9 (PMC5735092; doi:10.1038/s41598-017-17672-9)
Supplement: Supplementary file 1 — Supplementary Table S1 [file 41598_2017_17672_MOESM1_ESM.pdf]

Novel inhibitors targeting Venezuelan equine encephalitis virus capsid protein identified using *In Silico* Structure-Based-Drug-Design

Sharon Shechter[x1+], David R. Thomas*[1], Lindsay Lundberg[o], Chelsea Pinkham[o], Shih-Chao Lin[o], Kylie M. Wagstaff*, Aaron Debono[#], Kylene Kehn-Hall[o] and David A. Jans*[+]

[x]Shechter Computational Solutions, Andover, MA, USA
*Nuclear Signaling Laboratory, Department of Biochemistry and Molecular Biology School of Biomedical Sciences, Monash University, Melbourne, Australia
[o]National Center for Biodefense and Infectious Diseases, School of Systems Biology, George Mason University, Manassas, VA, USA
[#]Monash Institute of Pharmaceutical Sciences, Parkville, Victoria, Australia

[1]These authors contributed equally to this work.

[+]To whom correspondence should be addressed,
S. Shechter, c/- Shechter Computational Solutions, 198 River Road, Andover, MA 01810 USA; email Shechter.solutions@gmail.com; Tel. 1+ 978-409-2945
OR
D.A. Jans, c/- DBMB, Room 146 Building 77, Monash Uni., Vic 3800, Australia; email david.jans@monash.edu; Tel. 61+ 3 99029341

Supplementary Table S1. Human proteins containing the KKPK sequence. Nuclear proteins containing the min-NLS sequence (KKPK) were identified from NLSdb[51] and LocSigBD[52] databases, in addition to literature search using Google Scholar.

Protein	NLS*
Fibroblast growth factor 1 (FGF1)	Confirmed[42,43]
TATA-box binding protein associated factor 8 (TAF8)	Confirmed[44]
Histone H1.0	Confirmed[45]
Immortalization up-regulated protein 1 (IMUP-1)	Confirmed[46,47]
Brefeldin A-inhibited guanine nucleotide-exchange protein 1 (BIG1)	Confirmed[48]
SRY-box 11 (Sox11)	Confirmed[49]
Heat shock protein 105 kDa (Hsp105)	Confirmed[50]
SIN3 transcription regulator family member A (SIN3A)	Putative[51]
Protein kinase C-binding protein 1 (PRKCBP1)	Putative[51]
SUZ12 polycomb repressive complex 2 subunit (SUZ12)	Putative[51]
PNN interacting serine and arginine rich protein (PNISR)	Putative[51]
E3 ubiquitin-protein ligase (TRIP12)	Putative[51]
Phosphorylated CTD-interacting factor 1 (PCIF1)	Putative[51]

* Confirmed denotes that the NLS has been experimentally validated, as opposed to predicted (“Putative”).

References

42 Zhan, X., Hu, X., Friedman, S. & Maciag, T. Analysis of endogenous and exogenous nuclear translocation of fibroblast growth factor-1 in NIH 3T3 cells. *Biochem Biophys Res Commun* **188**, 982-991 (1992).

43 Zhen, Y. *et al.* Nuclear import of exogenous FGF1 requires the ER-protein LRRC59 and the importins Kpnalpha1 and Kpnbeta1. *Traffic* **13**, 650-664, doi:10.1111/j.1600-0854.2012.01341.x (2012).

44 Soutoglou, E. *et al.* The nuclear import of TAF10 is regulated by one of its three histone fold domain-containing interaction partners. *Mol Cell Biol* **25**, 4092-4104, doi:10.1128/MCB.25.10.4092-4104.2005 (2005).

45 Schwamborn, K., Albig, W. & Doenecke, D. The histone H1(0) contains multiple sequence elements for nuclear targeting. *Exp Cell Res* **244**, 206-217 (1998).

46 Itoh, H. *et al.* Identification of hepatocyte growth factor activator inhibitor type 2 (HAI-2)-related small peptide (H2RSP): its nuclear localization and generation of chimeric mRNA transcribed from both HAI-2 and H2RSP genes. *Biochem Biophys Res Commun* **288**, 390-399, doi:10.1006/bbrc.2001.5767 (2001).

47 Kim, J. K., Ryll, R., Ishizuka, Y. & Kato, S. Identification of cDNAs encoding two novel nuclear proteins, IMUP-1 and IMUP-2, upregulated in SV40-immortalized human fibroblasts. *Gene* **257**, 327-334 (2000).

48 Citterio, C. *et al.* Effect of protein kinase A on accumulation of brefeldin A-inhibited guanine nucleotide-exchange protein 1 (BIG1) in HepG2 cell nuclei. *Proc Natl Acad Sci U S A* **103**, 2683-2688, doi:10.1073/pnas.0510571103 (2006).

49 Waldron-Roby, E. *et al.* Sox11 Reduces Caspase-6 Cleavage and Activity. *PLoS One* **10**, e0141439, doi:10.1371/journal.pone.0141439 (2015).

50 Saito, Y., Yamagishi, N. & Hatayama, T. Different localization of Hsp105 family proteins in mammalian cells. *Exp Cell Res* **313**, 3707-3717, doi:10.1016/j.yexcr.2007.06.009 (2007).

51 Nair, R., Carter, P. & Rost, B. NLSdb: database of nuclear localization signals. *Nucleic Acids Res* **31**, 397-399, doi:10.1093/nar/gkg001 (2003).

52 Negi, S., Pandey, S., Srinivasan, S. M., Mohammed, A. & Guda, C. LocSigDB: a database of protein localization signals. *Database (Oxford)* **2015**, doi:10.1093/database/bav003 (2015)
